# Disease Trends in Children and Adolescents in Japan: A Retrospective Observational Study Using the Nationwide Claims Data for 2012–2016

**DOI:** 10.3390/children11010081

**Published:** 2024-01-10

**Authors:** Maiko Suto, Kenji Takehara, Naho Morisaki, Akinori Moriichi, Ruoyan Gai, Rintaro Mori

**Affiliations:** 1 Department of Health Policy, National Center for Child Health and Development, 10-1 Okura 2-chome, Setagaya, Tokyo 157-8535, Japan; suto-ma@ncchd.go.jp; 2 Department of Social Medicine, National Center for Child Health and Development, 10-1 Okura 2-chome, Setagaya, Tokyo 157-8535, Japan; morisaki-n@ncchd.go.jp; 3 Division of Specific Paediatric Chronic Disease Information, National Center for Child Health and Development, 10-1 Okura 2-chome, Setagaya, Tokyo 157-8535, Japan; moriichi-a@ncchd.go.jp; 4 Department of Empirical Social Security Research, National Institute of Population and Social Security Research, 2-2-3, Uchisaiwaicyo, Chiyoda-ku, Tokyo 100-0011, Japan; gai-ruoyan@ipss.go.jp; 5 Health Policy for Children and Families, Graduate School of Medicine, Kyoto University, Yoshida-Konoe-cho, Sakyo-ku, Kyoto 606-8501, Japan; rintaromori@gmail.com

**Keywords:** child health, adolescents, claims data, National Database of Health Insurance Claims of Japan, Japan

## Abstract

This study aimed to clarify diseases that occur more frequently by age and identify the peaks and trends of each disease from infancy to adolescence for early detection and treatment. This retrospective observational study was conducted using Japan’s National Database of Health Insurance Claims Specific Health Checkups from January 2012 to December 2016. Using peak ages and trends in the number of patients, we grouped diseases by the International Classification of Diseases chapters. Although diseases that peaked during infancy were the most common (10 disease chapters), other diseases peaked at school-going age and adolescence. Diseases in four chapters peaked during adolescence and continued to increase toward the age of 18. These four chapters included mental, behavioral, and neurodevelopmental disorders; diseases of the nervous system; the genitourinary system; and pregnancy, childbirth, and the puerperium. Childhood-onset diseases can affect long-term health and healthcare needs, and timely screening and guidance based on disease trends can provide an effective intervention. To establish a child healthcare system that provides preventive support for children and adolescents’ physical, psychological, and social health, further research is needed to comprehensively understand the issues per age and developmental stage.

## 1. Introduction

Globally, the number of deaths caused by infectious diseases and nutritional and neonatal disorders is declining; however, non-communicable diseases and injuries are becoming increasingly prevalent in children and adolescents [1]. More than 2.1 billion children and adolescents under the age of 20 are affected by non-communicable diseases, including mental health disorders [2]. Unintentional injuries are the leading cause of death, with more than 1600 children and adolescents under the age of 19 dying each day from preventable injuries [3]. The risk factors of non-communicable diseases and behavioral patterns that lead to disease often emerge during childhood and adolescence; consequently, it is vital to address these issues earlier in life [2,4,5]. In order to establish appropriate child healthcare systems for prevention and early detection, there is an urgent need to understand changing trends in disease burden at each developmental stage.

“Bright Futures” is a well-known guideline developed by the American Academy of Pediatrics for annual health screening based on evidence-informed priorities for children for each age [6,7]; however, there is still insufficient evidence from high-quality research in pediatric primary-care settings. This is due to a lack of adequate evidence for preventive services for children compared to those for adults [8]. Additionally, the leading causes of death and disability may vary by region [1,9]. Therefore, accumulating more evidence to understand the disease burden of children and adolescents across countries and regions is an important issue.

Health insurance claims are one of the resources that provide a comprehensive understanding of children’s health issues. Japan has a universal health insurance system, and the National Database of Health Insurance Claims Specific Health Checkups of Japan (NDB) includes most health insurance claims in Japan [10]. The NDB includes the diagnoses provided by physicians and can be used to describe disease trends and patterns. In Japan, several studies have been conducted on a national level to describe specific diseases in children, such as the number of children diagnosed with cerebral palsy [11] and the number of newborns undergoing phototherapy for neonatal hyperbilirubinemia [12]. However, to the authors’ knowledge, there are no Japanese studies that describe the frequency of diseases across a wide range of diseases in children and adolescents.

Therefore, this study clarified diseases that are more frequent by age and identified the peaks and trends of each disease from infancy to adolescence using the NDB. Through this research, we seek to contribute to a comprehensive understanding of children’s health issues and establish a healthcare system for the early detection and treatment of such diseases.

## 2. Methods

### 2.1. Study Design and Setting

We conducted a retrospective observational study using the NDB, which stores nationwide electronic health insurance claims data collected from medical institutes throughout Japan. The NDB’s health insurance claims data include hospital information (prefectures, number of beds, etc.), patient information (sex, age, etc.), and information on diseases (name, etc.), medical care, drugs administered, and medical fees. The NDB has been available for research since 2011. As of April 2016, electronic health insurance claims account for 98.0% of all claims (based on the total number of claims) [13]. Information on medical care not reimbursed by health insurance is not recorded in the NDB (e.g., the claims of welfare recipients, traffic accidents, injury to others, and homicide; these cases are covered by public funding, mandatory vehicle liability insurance, or compensation for damage). 

The Ministry of Health, Labour, and Welfare’s expert committee approved this research and confirmed compliance with the security requirements for using the NDB (approval number: 0168). Additionally, the Ethics Committee of the National Center for Child Health and Development (approval number: 1683) also approved this study in 2020.

#### Study Population and Data Source

We extracted and analyzed all health insurance claims data from the NDB, including inpatient/outpatient insurance claims data, diagnosis procedure combination data, and dental insurance claims data from January 2012 to December 2016 for patients aged zero to 18. 

### 2.2. Variables and Data Description

The NDB data contain anonymized IDs, and these IDs can be used to link the claims data for the same patient. In this study, we defined the frequency of patient IDs as the number of pediatric patients. The NDB displays two types of patient IDs: those generated using insurance numbers (ID1) and those generated using patient names (ID2). This study used ID1. We counted the number of pediatric patients by disease category based on the tenth revision of the International Classification of Diseases (ICD-10) block codes (called “intermediate classification” in Japan), patient ID, child age (the age of first appearance in the year applied), and year of diagnosis (January 2012 to December 2016). The NDB includes physicians’ preliminary diagnosis (categorized under “Suspected disease”) prior to the final diagnosis, which we excluded from the analysis.

### 2.3. Data Analysis

We listed the top five ICD-10 blocks by age based on the number of pediatric patients. We then summed the number of patients by ICD-10 block for each chapter classification. Next, we described the mean, maximum, and minimum values based on five-year averages by age and identified the peak age with the highest number of patients for each chapter classification. We excluded the following chapters related to aspects other than diseases: “External causes of morbidity” [V01-Y98], “Factors influencing health status and contact with health services” [Z00-Z99], and “Codes for special purposes” [U00-U89]. Two researchers discussed and created a disease group by ICD chapter classification based on peak age and patient ID frequency trends.

## 3. Results

As shown in Table 1, the frequency of “Acute upper respiratory infections” [ICD-10: J00-06] was ranked either first (0–17 years) or second (18 years). “Dermatitis and eczema” [L20-30] at 0–1 years, “Other acute lower respiratory infections” [J20-22] at 2–4 years, “Diseases of the oral cavity and salivary glands” [K00-14] at 5–10 years, “Other diseases of upper respiratory tract” [J30-39] at 11–14 years, and “Disorders of ocular muscles, binocular movement, accommodation and refraction” [H49-52] at 15–17 years (ranked highest at 18 years) were also ranked higher in each developmental stage.

The five disease groups were categorized into the following groups based on peak ages and trends: 

Group 1: The number of ICD-10 codes that peaked in infancy (0–1 years) and then gradually decreased included “Certain infectious and parasitic diseases” [A00-B99], “Diseases of the ear and mastoid process” [H60-H95], “Diseases of the respiratory system” [J00-J99], “Diseases of the skin and subcutaneous tissue” [L00-L99], “Certain conditions originating in the perinatal period” [P00-P96], “Congenital malformations, deformations, and chromosomal abnormalities” [Q00-Q99], and “Symptoms, signs, and abnormal clinical and laboratory findings not elsewhere classified” [R00-R99].

Group 2: The number of ICD-10 codes that peaked in infancy and gradually increased later included “Neoplasms” [C00-D48] (the number of “Benign neoplasms” [D10-36] is highest per age), “Diseases of the blood and blood-forming organs and certain disorders involving the immune mechanism” [D50-D89], and “Endocrine, nutritional and metabolic diseases” [E00-E90].

Group 3: The number of ICD-10 codes that peaked at school-going age (6–12 years) included “Diseases of the eye and adnexa” [H00-H59] (peak age: 9) and “Diseases of the digestive system” [K00-K93] (peak age: 6) (including the ICD-10 intermediate classification for “Diseases of the oral cavity and salivary glands”).

Group 4: The number of ICD-10 codes that peaked in adolescence (13–18 years) and decreased toward age 18 included “Diseases of the circulatory system” [I00-I99] (peak age: 15), “Diseases of the musculoskeletal system and connective tissue” [M00-M99] (peak age: 13), and “Injury, poisoning, and certain other consequences of external causes” [S00-T98] (peak age: 13).

Group 5: The number of ICD-10 codes that peaked in adolescence and increased toward age 18 included “Mental, behavioral, and neurodevelopmental disorders” [F00-F99] (peak age: 18), “Diseases of the nervous system” [G00-G99] (peak age: 17), “Diseases of the genitourinary system” [N00-N99] (peak age: 18), and “Pregnancy, childbirth, and the puerperium” [O00-O99] (peak age: 18 for maternal information).

**Figure 1:**
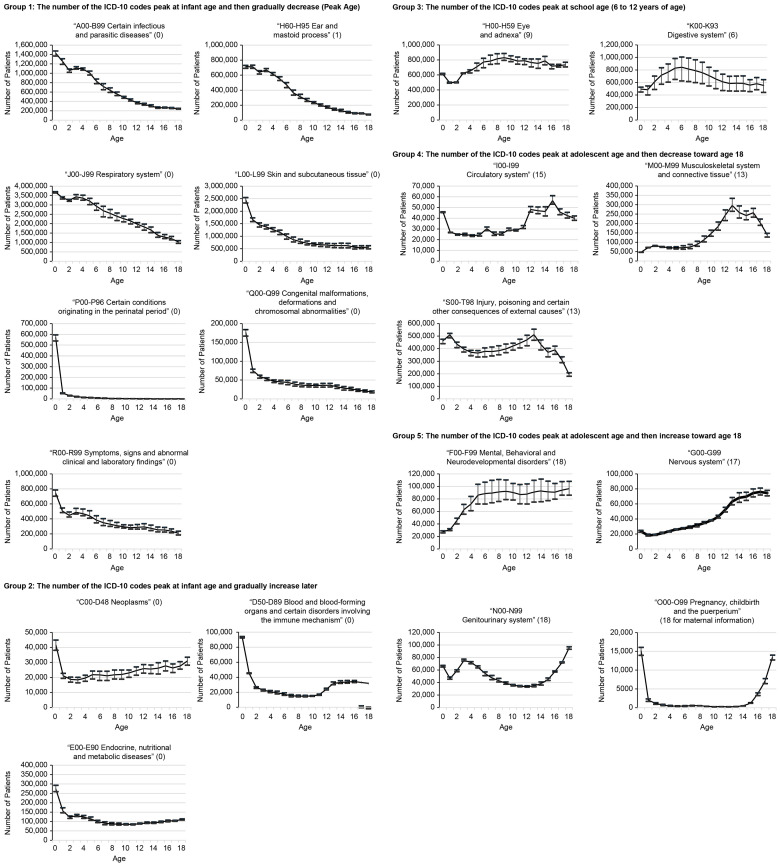
Disease trends by age (summed based on the ICD-10 block codes for each ICD chapter). Mean, maximum, and minimum values for five-year averages from 2012 to 2016.

The five categories are represented in Figure 1.

Even within the same ICD-10 chapter classification, disease peaks and trends differed when analyzed in greater detail based on ICD-10 block codes. To provide more detailed information on the number of pediatric patients among infants, children, and adolescents, the mean frequencies of the five-year averages (2012–2016) by patient ID using ICD-10 block codes are shown in an additional Excel file (see Supplementary Table S1). 

## 4. Discussion

This study described the number of pediatric patients by ICD-10 block code using health insurance claims data from the NDB in Japan. The disease frequency per age showed that respiratory diseases were the most common and that several disease groups peaked in infancy and decreased over time and into adolescence. Furthermore, using nationally representative data for Japan, this study’s findings highlight that in the case of some diseases, such as mental, behavioral, and neurodevelopmental disorders, and diseases of the nervous system, frequencies consistently increased toward adolescence.

For the most common diseases in children and adolescents in Japan, “Acute upper respiratory infections” were ranked highest among children and adolescents from 0 to 17 years of age and second among adolescents at 18 years of age. The chapter classification showed that several pediatric patients had infectious and parasitic diseases, diseases of the respiratory system, skin and subcutaneous tissue, eye and adnexa, and digestive system (including dental disease). In addition, the diseases of the oral cavity and salivary glands in children of school-going age, and disorders of the ocular muscles, binocular movement, accommodation, and refraction in adolescents also had high rankings in the list of diseases in the health insurance claims data. These results on the frequency of diseases are similar to the findings of other Japanese studies based on national statistics with a high level of representativeness [14,15]. 

In the 2017 Patient Survey conducted by the Ministry of Health, Labour, and Welfare, the frequencies of the following diseases were ranked higher among children aged 0 to 19 years: “diseases of the respiratory system”, “diseases of the digestive system” (including dental disease), and “diseases of the skin and subcutaneous tissue” [14]. An analysis of the prevalence of diseases and abnormalities in the 2019 School Health Statistics Research conducted by the Ministry of Education, Culture, Sports, Science, and Technology identified “dental caries” as the most common disease in kindergarten and elementary school children, followed by “uncorrected visual acuity of <1.0”. The analysis also identified “uncorrected visual acuity of <1.0” as the most common disease in junior high and high school children, followed by “dental caries” [15]. As the School Health Statistics Research was based on the results of school health examinations, the survey did not include items related to temporary diseases, health conditions, or abnormalities such as influenza, rhinitis, and pharyngitis due to a common cold. Infectious diseases, respiratory diseases, and skin diseases are also recognized as common childhood illnesses in other countries [16]. With regard to ophthalmologic and dental diseases, the large number of patients may be attributed to school health checkups, since ophthalmologic and dental checkups are conducted annually at schools in Japan, and if necessary, hospital visits are recommended. Although some of these common diseases may be mild, routine examination and management are important to prevent serious conditions. 

Regarding the peak age for each disease classification, diseases that peaked during infancy were the most common. Some diseases also peaked in adolescence and continued to increase toward age 18. Diseases that peaked in adolescence included “Mental, behavioral and neurodevelopmental disorders”, “Diseases of the nervous system”, “Diseases of the genitourinary system”, and diseases related to “Pregnancy, childbirth, and the puerperium”; however, routine screening for these diseases is not available for children and adolescents in Japan, which suggests that there is limited scope for primary prevention and early detection. Globally, the peak age for the onset of several mental disorders is reported to be 14.5 years and the median age is 18 years [17]. The World Health Organization’s (WHO) Global Health Estimates (GHE) provide global data on death and disability by region and country, as well as by age group and cause [18], but there is still insufficient information regarding the trends and patterns for various diseases per age and developmental stage in children and adolescents. Results are often clustered into age groups, such as every five years; therefore, the diseases common per age are not well understood and the preventable health issues may not be properly addressed.

Globally, mental health is considered a major adolescent health issue. Over 13% of adolescents aged 10 to 19 years old are estimated to live with a diagnosed mental disorder [19]. Studies have also indicated that 50% of mental health disorders are established by the age of 14 years, and the longer the duration of untreated mental health problems, the greater the long-term burden of disease [20]. Many children and adolescents do not receive appropriate care due in part to misconceptions and prejudice, although the importance of early detection of mental health problems is well documented [19]. Some studies have shown that prevention programs, such as those based on cognitive behavioral therapy, are effective in reducing children’s mental health burden [21,22]. In addition to physical illnesses, which are often the primary focus of school health examinations, children’s mental and behavioral problems, such as depression, eating disorders, sleep problems, alcohol, tobacco and illicit drug use, and risky sexual behaviors [23], also need to be addressed.

Childhood diseases associated with an age-related increase in incidence frequency, such as eye and dental diseases or mental illnesses, usually persist for a long period of time and even into adulthood. Preventive interventions and screenings in children and adolescents may lead to a long-term reduction in the disease burden. 

The establishment of a healthcare system that includes regular checkups and counseling in schools or primary care settings for the early detection and treatment of mental health and behavior problems among school-age children and adolescents is essential. The American Academy of Pediatrics’ “Bright Futures” guidelines help healthcare providers to anticipate problems that children may face and recommend preventative screenings. These guidelines further emphasize that for anticipatory guidance to be effective, interventions must be timely, that is, provided at the appropriate age [23]. Further research is needed to comprehensively understand health issues in the context of a child’s age and developmental stage. It is also necessary to establish a child healthcare system that can provide timely screening and guidance based on peak age frequency trends in childhood diseases, and interventions should focus on the period shortly before the estimated peak age and during the peak age. In the future, it is also desirable to conduct research that analyzes the effects of preventive programs and policies and to improve the use of comprehensive healthcare data on children and adolescents.

### Study Limitations

In this study, we defined the frequency of patient ID in health claims data as the number of pediatric patients; however, there are certain limitations to this definition. First, the number of patients was overestimated because we used patient IDs generated based on insurance numbers to count patients. These patient IDs changed in response to patients’ participation in or withdrawal from insurance policies due to their parents’ life events such as job changes, divorce, or death. This led to duplicate counting since one patient could be identified more than once [24]. In this study, we retrieved approximately 1.3 million patient IDs with eczema and respiratory disease at age 0—the highest number of IDs in our study. In contrast, the number of births in Japan from 2012 to 2016 ranged from 1,037,232 (2012) to 977,242 (2016) [25]. This difference between the two sets of figures points to duplicate counting; however, in the context of this study, duplicate counting occurred equally in all diseases and should not inflate the number of specific cases. It is also important to note that many municipalities have introduced a medical subsidy for preschool children; therefore, it is likely that medical service utilization has increased significantly for younger children, especially children in the preschool category. 

Second, this study did not evaluate the validity of the diagnoses. The NDB database is based on insurance claims’ needs; therefore, the disease name does not necessarily reflect patients’ actual medical diagnoses. In some studies that used the NDB, diseases were defined to combine the name of the disease with specific types of medical care when health insurance claims data were analyzed. 

Third, the NDB does not record data on medical care not reimbursed by health insurance. Data on medical care fully covered by public funding (e.g., for those of welfare recipients) and covered by mandatory vehicle liability insurance or compensation for damages (e.g., traffic accidents, injury to others, and homicide) were not included in the analysis in this study. Since traffic accidents are a leading cause of death among children and adolescents [26], the absence of such data would have skewed disease trends. In addition, accurate mortality data are not currently recorded in the NDB, and mortality trends were not analyzed in this study. 

Finally, while this study attempted to identify overall trends for policy recommendations in the child healthcare system, future analyses of disease trends based on a more detailed disease classification than ICD-10 block codes and chapter classifications are also needed from a clinical perspective.

## 5. Conclusions

Regardless of the above limitations, the NDB covers most medical care in hospitals throughout Japan. Therefore, it is a useful data source for a comprehensive analysis of the healthcare needs of children who require intervention. The results of this study revealed that some diseases, such as mental, behavioral, and neurodevelopmental disorders, diseases of the nervous system, the genitourinary system, and pregnancy, childbirth, and puerperium tend to increase in children of school-going age and among adolescents, indicating a need for improved screening and greater access to care for these issues in children based on their developmental stage. Further research on monitoring health issues and developing early preventive interventions is essential to establish a comprehensive and seamless support system that addresses children’s physical, psychological, and social health needs. In addition, future research that analyzes the effectiveness of prevention programs and policies and focuses on improving the use of comprehensive medical data on children and adolescents is also desirable.

## Figures and Tables

**Table 1:** Ranking of the number of patients for each ICD-10 block code by age (created using the five-year average from 2012 to 2016).

	1st	ICD Code	2nd	ICD Code	3rd	ICD Code	4th	ICD Code	5th	ICD Code
Age_0	Acute upper respiratory infections	J00-06	Dermatitis and eczema	L20-30	Other acute lower respiratory infections	J20-22	Intestinal infectious diseases	A00-09	Chronic lower respiratory diseases	J40-47
Age_1	Acute upper respiratory infections	J00-06	Dermatitis and eczema	L20-30	Other acute lower respiratory infections	J20-22	Intestinal infectious diseases	A00-09	Chronic lower respiratory diseases	J40-47
Age_2	Acute upper respiratory infections	J00-06	Other acute lower respiratory infections	J20-22	Chronic lower respiratory diseases	J40-47	Dermatitis and eczema	L20-30	Other diseases of upper respiratory tract	J30-39
Age_3	Acute upper respiratory infections	J00-06	Other acute lower respiratory infections	J20-22	Chronic lower respiratory diseases	J40-47	Other diseases of upper respiratory tract	J30-39	Dermatitis and eczema	L20-30
Age_4	Acute upper respiratory infections	J00-06	Other acute lower respiratory infections	J20-22	Chronic lower respiratory diseases	J40-47	Other diseases of upper respiratory tract	J30-39	Diseases of oral cavity and salivary glands	K00-14
Age_5	Acute upper respiratory infections	J00-06	Diseases of oral cavity and salivary glands	K00-14	Other diseases of upper respiratory tract	J30-39	Chronic lower respiratory diseases	J40-47	Other acute lower respiratory infections	J20-22
Age_6	Acute upper respiratory infections	J00-06	Diseases of oral cavity and salivary glands	K00-14	Other diseases of upper respiratory tract	J30-39	Chronic lower respiratory diseases	J40-47	Other acute lower respiratory infections	J20-22
Age_7	Acute upper respiratory infections	J00-06	Diseases of oral cavity and salivary glands	K00-14	Other diseases of upper respiratory tract	J30-39	Chronic lower respiratory diseases	J40-47	Other acute lower respiratory infections	J20-22
Age_8	Acute upper respiratory infections	J00-06	Diseases of oral cavity and salivary glands	K00-14	Other diseases of upper respiratory tract	J30-39	Other acute lower respiratory infections	J20-22	Chronic lower respiratory diseases	J40-47
Age_9	Acute upper respiratory infections	J00-06	Diseases of oral cavity and salivary glands	K00-14	Other diseases of upper respiratory tract	J30-39	Other acute lower respiratory infections	J20-22	Chronic lower respiratory diseases	J40-47
Age_10	Acute upper respiratory infections	J00-06	Diseases of oral cavity and salivary glands	K00-14	Other diseases of upper respiratory tract	J30-39	Other acute lower respiratory infections	J20-22	Chronic lower respiratory diseases	J40-47
Age_11	Acute upper respiratory infections	J00-06	Other diseases of upper respiratory tract	J30-39	Diseases of oral cavity and salivary glands	K00-14	Other acute lower respiratory infections	J20-22	Disorders of ocular muscles, binocular movement, accommodation, and refraction	H49-52
Age_12	Acute upper respiratory infections	J00-06	Other diseases of upper respiratory tract	J30-39	Diseases of oral cavity and salivary glands	K00-14	Disorders of ocular muscles, binocular movement, accommodation, and refraction	H49-52	Other acute lower respiratory infections	J20-22
Age_13	Acute upper respiratory infections	J00-06	Other diseases of upper respiratory tract	J30-39	Diseases of oral cavity and salivary glands	K00-14	Disorders of ocular muscles, binocular movement, accommodation, and refraction	H49-52	Other acute lower respiratory infections	J20-22
Age_14	Acute upper respiratory infections	J00-06	Other diseases of upper respiratory tract	J30-39	Diseases of oral cavity and salivary glands	K00-14	Disorders of ocular muscles, binocular movement, accommodation, and refraction	H49-52	Other acute lower respiratory infections	J20-22
Age_15	Acute upper respiratory infections	J00-06	Disorders of ocular muscles, binocular movement, accommodation, and refraction	H49-52	Other diseases of upper respiratory tract	J30-39	Diseases of oral cavity and salivary glands	K00-14	Other acute lower respiratory infections	J20-22
Age_16	Acute upper respiratory infections	J00-06	Disorders of ocular muscles, binocular movement, accommodation, and refraction	H49-52	Other diseases of upper respiratory tract	J30-39	Diseases of oral cavity and salivary glands	K00-14	Other acute lower respiratory infections	J20-22
Age_17	Acute upper respiratory infections	J00-06	Disorders of ocular muscles, binocular movement, accommodation, and refraction	H49-52	Diseases of oral cavity and salivary glands	K00-14	Other diseases of upper respiratory tract	J30-39	Dermatitis and eczema	L20-30
Age_18	Disorders of ocular muscles, binocular movement, accommodation, and refraction	H49-52	Acute upper respiratory infections	J00-06	Diseases of oral cavity and salivary glands	K00-14	Other diseases of upper respiratory tract	J30-39	Dermatitis and eczema	L20-30

## Data Availability

This published article and its supplementary information files include all data generated or analyzed during this study.

## Supplementary Materials

The following supporting information can be downloaded at: https://www.mdpi.com/article/10.3390/children11010081/s1, Table S1: Number of patients for each ICD-10 block code by age.

## Abbreviations

NDB: National Database of Health Insurance Claims Specific Health Checkups of Japan; ICD-10: International Classification of Diseases, 10th revision (ICD-10).
